# Post-operative antibiotics for cutaneous abscess after incision and drainage: Variations in clinical practice

**DOI:** 10.1099/acmi.0.000441

**Published:** 2022-10-28

**Authors:** Michael El Boghdady, Béatrice Marianne Ewalds-Kvist, Sarah Zhao, Ahmad Najdawi, Aggelos Laliotis

**Affiliations:** ^1^​ Department of General Surgery, Croydon University Hospital, London, UK; ^2^​ The University of Edinburgh, Edinburgh, UK; ^3^​ St. George's University of London, London; ^4^​ Stockholm University, Stockholm, Sweden; ^5^​ University of Turku, Turku, Finland

**Keywords:** Antibiotics, Cutaneous abscess, Microguidelines, Incision and drainage

## Abstract

**Background.:**

Acute cutaneous abscess is a common surgical condition that mostly requires incision and drainage. Despite this, there is no standardized national or international guidance on post-operative antibiotics prescription. Traditionally, antibiotics are not indicated unless complications and/or risk factors such as immunocompromisation, diabetes or cellulitis exist. We aimed to study the local practice for post-operative antibiotics prescription for cutaneous abscesses in a UK university teaching hospital.

**Methods.:**

Retrospective data collection for emergency general surgical admissions for a period of 6 months was carried out. All patients with cutaneous abscesses were included in this analysis. Scrotal, breast and limb abscesses were excluded. Patients’ demographics, co-morbidities and complications, including local (cellulitis, necrosis) and systemic (e.g sepsis), were studied. Approval for access to patient data was granted by the local clinical governance department prior to the commencement of this study. Computations were performed using IBM SPSS version 26. Chi square (*X* ^2^), Pearson correlation (*r*), one or two samples *t*-test (one or two tailed) were applied.

**Results.:**

A total of 148 patients were included. The mean age was 40 years (55 % males). The most common site of abscess was perianal (27.7 %), followed by pilonidal (20.3 %) and axilla (16.9 %). A total of 107 (73 %) were managed surgically with incision and drainage, and of these 92 (86 %) were managed within 24 h. Altogether, 83 (76 %) were prescribed post-operative antibiotics, while only 25 (23 %) had indications. The most used post-operative empirical antibiotics was co-amoxiclav (59 %). There was a significant relationship between ‘abscess site’ × ‘antibiotics’ [*X* ^2^ (36)=54.8, *P*=0.023]. A total of 103 patients’ average duration of post-operative antibiotics was 7.2 (sd 2.9) days. Ten patients subject to readmission spent an average of 8.4 (sd 3.8) days on antibiotics.

**Conclusions.:**

There were variations in clinical practice regarding post-operative antibiotic prescription for cutaneous abscesses. Research is required in the future in cooperation with microbiologists to develop a standardized evidence-based treatment protocol for the management of such a common surgical condition.

## Background

Cutaneous abscess is known to be an isolated collection of pus within the dermis and deeper skin tissues. The term ‘skin and soft-tissue infection (SSTI)’ covers a range of pathological conditions involving the skin and underlying subcutaneous tissue, fascia, or muscle; SSTI varies from uncomplicated superficial infection to toxic necrotizing infection [1]. Necrotizing skin and soft-tissue infections (NSTIs) are life-threatening and incapacitating infections that constitute a diagnostic and therapeutic challenge. Early recognition of severe SSTIs such as NSTIs must rapidly be halted by broad-spectrum antibiotics and aggressive surgical debridement [2]. Purulent infection must be treated surgically with an incision and drainage for elimination of the infection [3].

To date, the role of adjuvant antibiotic therapy in addition to incision and drainage (I and D) has been controversial [4], mainly because previous randomized controlled trials (RCTs) have failed to consistently show benefit. Traditionally, antibiotics are indicated in certain local complications, such as cellulitis or skin necrosis, as well as in systemic complications or comorbidities such as diabetes mellitus, immunocompromising conditions, and sepsis.

On the other hand, there is currently limited national or international guidance on post-operative prescription of antibiotics for uncomplicated cutaneous abscesses. The Association of Coloproctology of Great Britain and Ireland (ACPGBI) recommended the incision and drainage of perianal abscesses within 24 hours, with no indication for post-operative antibiotics prescription for uncomplicated perianal abscesses [5]. A previous systematic review and meta-analysis concluded that antibiotics provide a modest reduction in the risk of treatment failure, recurrence, additional surgical procedures and hospitalization, but reduce pain during treatment [6]. However, the decision whether or not to use antibiotics should consider an individual patient’s clinical factors (e.g. severity of infection, immunocompromised state) and individual values and preferences [6]. There was likewise a published recommendation stating that patients who place a higher value on the possibility of avoiding abscess recurrence may choose clindamycin, while those who place a higher value on avoiding diarrhoea and on minimizing costs are likely to prefer trimethoprim/sulfamethoxazole [7].

We aimed to study the local practice for post-operative antibiotics prescription for cutaneous abscesses in a UK university teaching hospital.

## Methods

A retrospective study among all emergency general surgical admissions for patients with superficial cutaneous abscesses for a period of 6 months from July to December 2020 was carried out. Data were collected from the hospital electronic database.

Scrotal, breast and limb abscesses were excluded, as they were treated in different departments. Patients’ demographics, co-morbidities and complications, both local (such as cellulitis and necrosis) and systemic (such as diabetes, sepsis and immunocompromission), were studied.

### Statistical analysis

Statistical analysis was performed using IBM SPSS version 26. Pearson correlation (*r*) was used for a relationship for type of antibiotics before and after abscess surgery. One sample *t*-test was used to test significance between independent variables in the same sample. One or two-tailed *t*-tests were applied depending on hypothesis and relevance. Chi square (*X* ^2^) was used for testing the relationship between, e.g., ‘antibiotics’ and ‘abscess site’.

## Results

### Subjects

A total of 148 patients with cutaneous abscesses presented for emergency general surgical admissions during a 6-month period at a UK university teaching hospital. The patients’ mean age was 40 years (sd 16) but there was a gender difference: the 82 (55 %) males had a mean age of 42 years (sd 16.6), while the mean age of the 66 females was 36 years (sd 14.8) [*t* (145)=2.088, *P*=0.039].

### Abscess site

The most common three sites of abscess were perianal (27.7 %), followed by pilonidal (20.3 %) and axilla (16.9 %). Less frequent were groin (10.1 %) gluteal (9.5 %) neck/back (7.4 %) and other abscess sites (8.1 %). There was a significant difference within abscess sites [one-way *t* (147)=19.29, *P* <0.001]. Patients younger than 25 years had more pilonidal abscesses compared to those older than 52 years, with more perianal abscesses [*t* (58.5)=3058, *P* <0.003].

### Surgery and antibiotics prescription

**Fig. 1.**
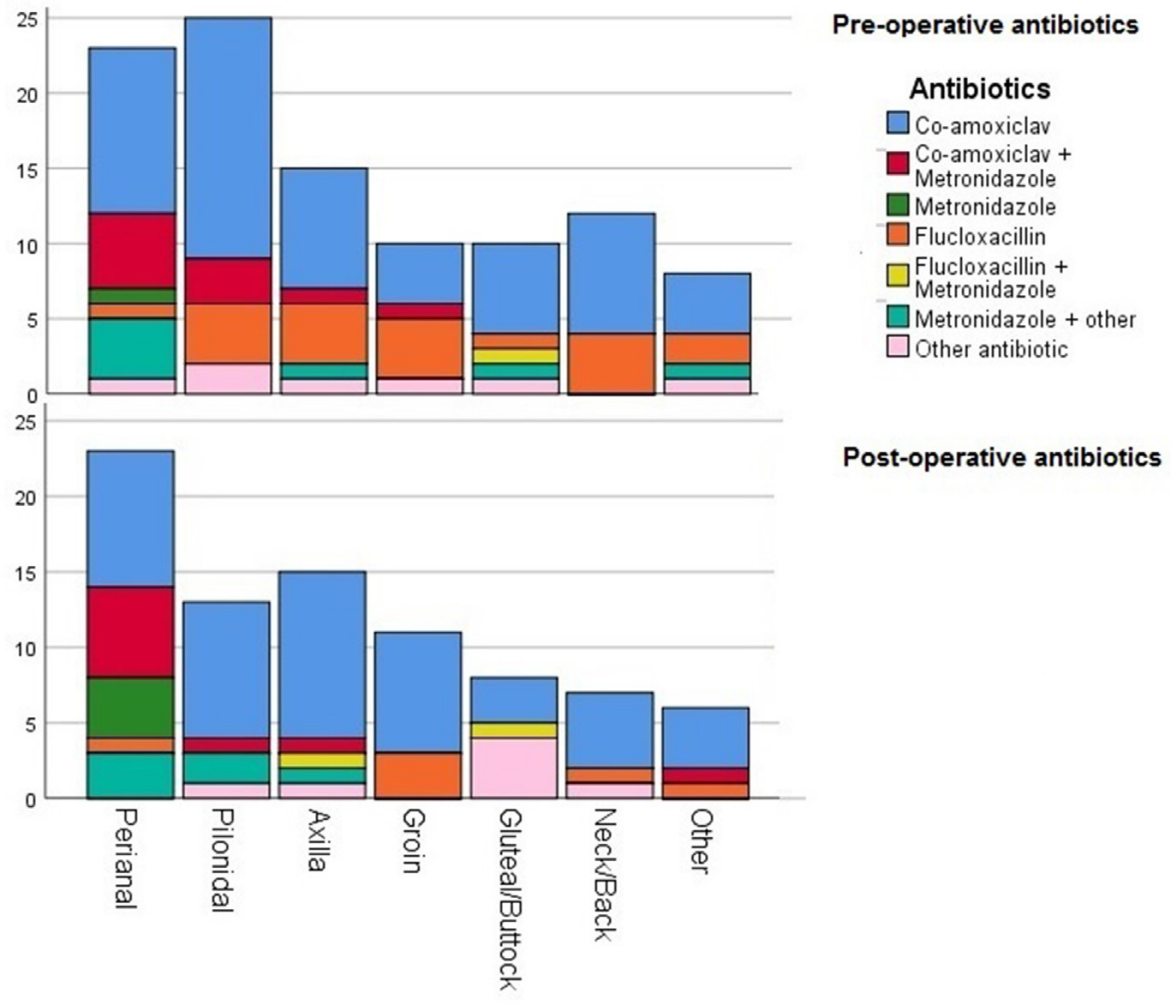
Pre- and post-operative antibiotic use in relation to the anatomical abscess site.

A total of 107 (73 %) were managed surgically with incision and drainage, and of these 92 (86 %) were managed within 24 h. Altogether, 83 (76 %) were prescribed antibiotics post-surgery. Eleven different antibiotics were prescribed pre-operatively (*n* of patients in parentheses): ceftriaxone (1), clarithromycin (3), co-amoxiclav (68), co-trimoxazole (2), doxycycline (3), flucloxacillin (24), linezolid (2), meropenem (1), metronidazole (21), tazocin (1) and teicoplanin (2) (Fig. 1; Table 1).

**Table 1.** Pre-and post-operative antibiotics with comorbidities, complications and readmissions

	Pre- operative	Post- operative	Complications	Readmission
**Comorbidities:** COVID-19, diabetes mellitus (1 and 2), immuno-compromission, sepsis **Complications:** cellulitis, erythema, skin necrosis, suspected necrotizing fasciitis				
Co-amoxiclav (amoxicillin/clavulanic acid	13	12	2	3
Co-amoxiclav *+metronidazole*	0	2		0
Flucloxacillin	3	1		2
Metronidazole +other	3	4	1	0
	Ceftriaxone, linezolid, meropenem, tazocin	Co- trimoxazole, ertapenem, daptomycin tazocin	Co- trimoxazole	
Other antibiotics	1	1	3	1
	Linezolid	Ciprofloxacin, clarithromycin clindamycin, doxycycline, flucloxacillin, tazocin, teicoplanin	Clindamycin, flucloxacilli tazocin, teicoplanin	Clindamycin, mupirocin
Unknown/no antibiotic	4	5	2	4
No. of patients	25	25	8	10

Post-operatively, 17 antibiotics were used; of these 9 had also been used pre-operatively [*r* (19)=0.98, *P* <0.001]. The following agents were post-operatively used: cefalexin (1), ciprofloxacin (1), clarithromycin (1), clindamycin (4), co-amoxiclav (59), co-trimoxazole (3, for immunocompromised patients), doxycycline (1), duptamycin (1), ertapenem (1), flucloxacillin (24), linezolid (2), metronidazole (21), mupirocin (1), tazocin (3, piperacillin/tazobactam) and teicoplanin (3) (Fig. 1; Table 1).

Co-amoxiclav (amoxicillin/clavulanic acid) is the most used antibiotic both pre-and post-operatively. There was a significant relationship between post-operative ‘antibiotics’ × ‘abscess site’ [*X* ^2^ (36)=54.8, *P*=0.023].

### Indications of antibiotics

**Fig. 2.**
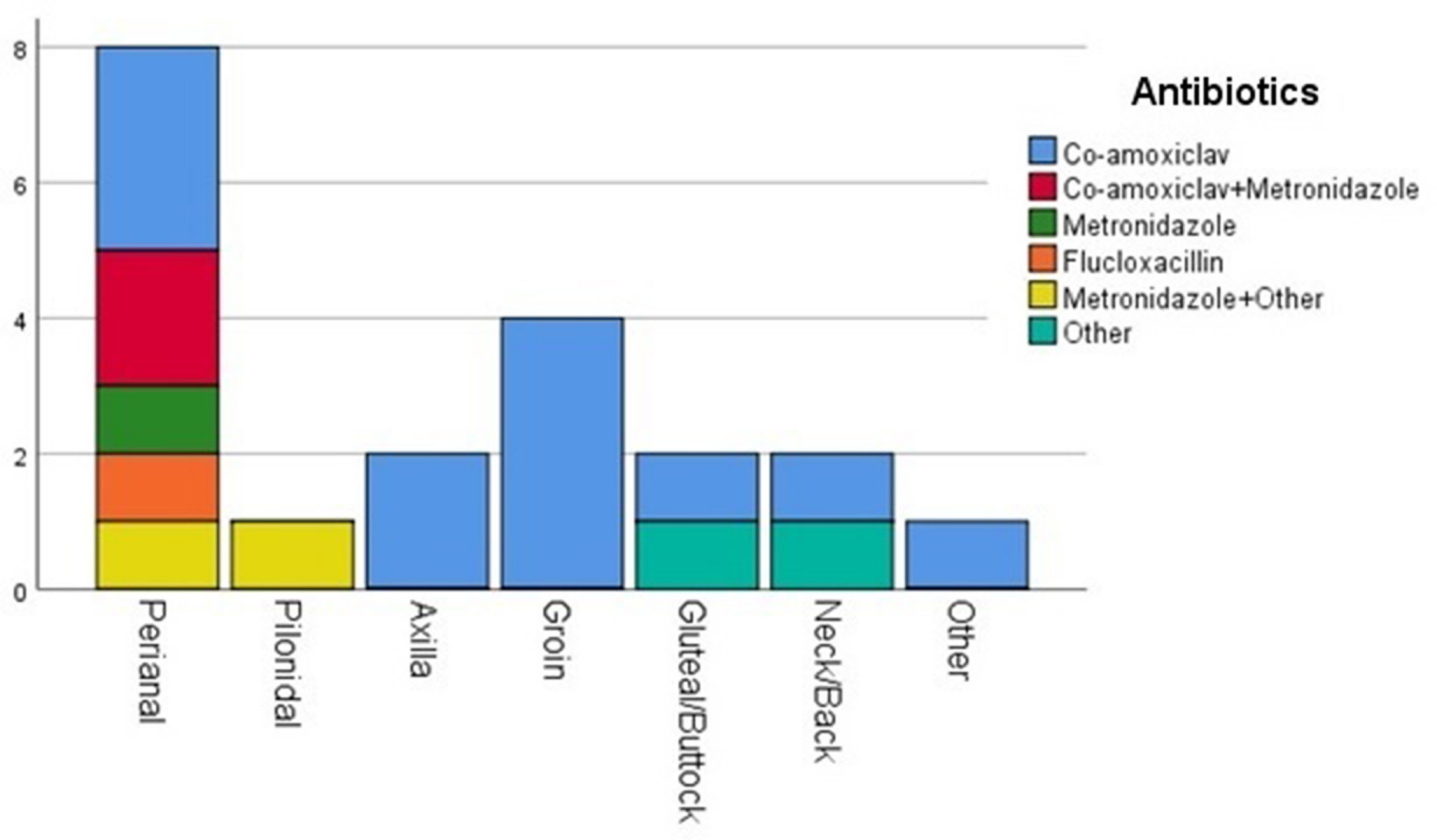
Post-operative antibiotics for patients with comorbidities who underwent drainage for cutaneous abscess .

Indications for antibiotics as well as implemented antibiotics and the abscess sites are presented in Table 1 and in Fig. 2. The one-sample *t*-tests between both abscess sites [*t* (24)=7.688, *P* <0.001] and antibiotics [*t* (19)=4.901, *P* <0.001] were significant. ‘Other antibiotics’ are shown in Table 1.

Out of 148 patients with cutaneous abscesses, 11 were allergic to penicillin and 2 to flucloxacillin. Two patients were pregnant out of 66 females and 1 of them was prescribed co-amoxiclav post-surgery. Altogether 103 patients’ average time on antibiotics was 7.2 (sd 2.9) days with a range 16 days. However, younger patients <25 years spent on average 6.4 (sd 3.1) days compared to older patients >52 years who spent on average 8.8 (sd 3.9 days) on antibiotics [*t* (41.8)=2 265, *P*=0.029). A total of 25 (16.9 %) patients suffered from local or systemic comorbidities and spent an average of 8.37 (sd 3.24) days [*t* (18)=11.274, *P* <0.001] on treatment. Eight (5.4 %) patients suffered from complications and spent an average of 8.33 (sd 3.20) days [*t* (6)=6.371, *P*=0.001] on treatment. Moreover, 10 (6.8 %) patients with readmission spent an average of 8.43 (3.78) days [*t (*6)=5.900, *P*=0.001) on treatment. Treatment related to recurrence comprised conservative management with antibiotics in 6 patients and incision and drainage in 10, and of these 7 patients received post-operative antibiotics.

## Discussion

To our knowledge, this is the first report to highlight the clinical variations in antibiotics prescription after the incision and drainage (I and D) of uncomplicated cutaneous abscesses. A total of 73% patients were managed surgically with I and D, of these 86 % were surgically managed within 24 hours. A total of 76 % were prescribed antibiotics post-operatively.

Altogether 11 different antibiotics were administered pre-operatively in our study, of these co-amoxiclav and metronidazole, combined or alone, were the most prescribed antibiotics. Post-operatively, 17 different antibiotics were used. Likewise, co-amoxiclav was the most commonly prescribed antibiotic, which was also combined with metronidazole in eight cases. Clindamycin and tazocin were each applied to four patients who suffered from comorbidities or complications in the form of erythema/cellulitis, suspected necrotizing fasciitis or recurrent abscess. This was in contrast to the BMJ recommendation for the prescription of trimethoprim/sulfamethoxazole or clindamycin after surgery [7].

Our patients’ most common comorbidity was diabetes mellitus (DM). Along with immunosuppressed patients, we envisage the prescription of antibiotics post-I and D for cutaneous abscesses in diabetic patients. It was revealed that there is a link between DM and infectious agents (viral, bacterial, fungal, parasitic, prion-like) [8]. In particular, people with DM who are predisposed to infections are subjected to a considerable intake of antimicrobials, enabling the selection of drug-resistant strains [9].

Local complications such as cellulitis and skin necrosis required antibiotics prescriptions, in addition to necrotizing soft tissue infections, which are a broad category of bacterial and fungal skin infections. For cases with suspected necrotizing fasciitis, clindamycin, tazocin and teicoplanin were more commonly prescribed. However, none of these patients were proven to have necrotizing fasciitis. A previous study recommended empirical antibiotics as soon as a diagnosis of NSTI is available, followed by a switch over to culture-guided broad-spectrum empirical antibiotics, covering Gram-positive (including methicillin-resistant *Staphylococcus aureus *, MRSA), Gram-negative and anaerobic bacteria [10]. However, in our study we noted that culture swabs were not sent routinely in our clinical practice, which is in agreement with reports concluding that microbiological results are often not correlated with the development of post-operative complications [11]. While the routine use of pus swabs is mainly suggested when clinical concerns arise; for example, in cases with recurrent perianal sepsis, immuno-compromised status or extensive soft tissue necrosis, especially when these features are associated with systemic sepsis [12].

In our study, the most common abscess sites were perianal, followed by pilonidal and axilla. This fact differed from a previous report that identified the back, axillae and groin as the most common sites of presentation over a 12-month period of admission [13]. Perianal site infection was mostly treated with amoxicillin/clavulanic acid alone or in combination with metronidazole (nitroimidazole) as an antiprotozoal agent. This finding is in contrast to the ACPGBI’s no-antibiotics recommendation in uncomplicated perianal abscesses after I and D [5]. An increased awareness of national and local guidelines is required for patients with perianal abscesses to improve antibiotic stewardship in these surgical patients and avoid unnecessary drugs prescriptions.

Regarding the duration of post-operative antibiotic prescription, 103 patients’ average time on antibiotics was 7.2 (sd 2.9) days with a range 16 days. Younger patients spent approximately 6,4 (sd 3.1) days on antibiotics compared to those older than 50 years, who spent 8.8 (sd 3.9 days) on antibiotics. Patients with local or systemic comorbidities spent approximately 8.4 (sd 3.24) days on treatment. A previous study noted a higher likelihood of cure with antibiotic courses beyond 5 or 7 days (up to 10), in agreement with our results [14].

One of the limitations of this study is its retrospective nature. However, all data were collected from our hospital’s electronic database. None of the patients needed long-term follow-up, except those who were readmitted with recurrence. A previous report pointed out that a retrospective cohort study, also called a historical study, is typically compiled from databases of healthcare records that have already been collected [15]. This means that there could have been several different persons who compiled the file with the diagnoses, surgical procedures and antibiotics, and we trust that their observations and comments are adequate. However, an advantage of this retrospective cohort study design is that exposure to risk factors in the forms of comorbidity, anaesthestic, I and D, and antibiotics are recorded before the occurrence of the outcome. This is crucial because it allows the temporal sequence of risk factors and outcomes to be assessed.

Local microbiological guidelines for antibiotics prescription after incision and drainage of cutaneous abscesses did not exist, and there was no national or international guidance. This highlights the significance of conducting such a study to reveal varying clinical practices and the need for urgent microbiology guidelines. The local department of microbiology was contacted regarding the results of this study and they are going to ratify this within their empirical guidance.

## Conclusion

This study has identified significant variation in clinical practice regarding post-operative antibiotic usage in superficial cutaneous abscesses. Research is required in the future in cooperation with microbiologists to develop a standardized evidence-based treatment protocol for the management of such a common surgical condition.
